# Human immunodeficiency virus seroconversion presenting with acute inflammatory demyelinating polyneuropathy: a case report

**DOI:** 10.1186/1752-1947-2-370

**Published:** 2008-12-04

**Authors:** Derek J Sloan, Andrew Nicolson, Alastair RO Miller, Nick J Beeching, Mike BJ Beadsworth

**Affiliations:** 1 Tropical and Infectious Disease Unit, 3Z Link Corridor, Royal Liverpool University Hospital, Prescot Street, Liverpool, UK, L7 8XP; 2 Liverpool School of Tropical Medicine, Pembroke Place, Liverpool, UK; 3 Walton Centre for Neurology and Neurosurgery NHS Trust, Lower Lane, Fazakerley, Liverpool, L9 7LJ, UK

## Abstract

**Introduction:**

Acute Human Immunodeficiency Virus infection is associated with a range of neurological conditions. Guillain-Barré syndrome is a rare presentation; acute inflammatory demyelinating polyneuropathy is the commonest form of Guillain-Barré syndrome. Acute inflammatory demyelinating polyneuropathy has occasionally been reported in acute Immunodeficiency Virus infection but little data exists on frequency, management and outcome.

**Case presentation:**

We describe an episode of Guillain-Barré syndrome presenting as acute inflammatory demyelinating polyneuropathy in a 30-year-old man testing positive for Immunodeficiency Virus, probably during acute seroconversion. Clinical suspicion was confirmed by cerebrospinal fluid analysis and nerve conduction studies. Rapid clinical deterioration prompted intravenous immunoglobulin therapy and early commencement of highly active anti-retroviral therapy. All symptoms resolved within nine weeks.

**Conclusion:**

Unusual neurological presentations in previously fit patients are an appropriate indication for Immunodeficiency-Virus testing. Highly active anti-retroviral therapy with adequate penetration of the central nervous system should be considered as an early intervention, alongside conventional therapies such as intravenous immunoglobulin.

## Introduction

Human Immunodeficiency Virus (HIV) infection is neurotropic and a wide array of neurological presentations has been described. Alongside common presentations of meningitis, encephalitis and peripheral neuropathies, Guillain-Barré syndrome (GBS) has rarely been reported. Fewer reports exist of the acute inflammatory demyelinating polyneuropathy (AIDP) variant of GBS presenting as an acute HIV seroconversion. Little data exists on patient outcomes or treatment options in HIV-GBS, including intravenous immunoglobulin (IVIg) and highly active anti-retroviral therapy (HAART). We discuss the clinical presentation, outcome and management including the current evidence base.

## Case presentation

In 2007 a previously well 30-year-old homosexual man presented with a 5 day history of progressive bilateral ascending lower limb weakness, preceded by a flu-like illness lasting for 2 weeks. Examination revealed normal tone but slightly reduced power at Medical Research Council (MRC) grading 4/5 [1]. Sensation was intact but he was areflexic in both legs. Plantar responses were flexor. The rest of the clinical examination was normal.

Cerebrospinal fluid (CSF) examination showed 26 leucocytes/mm^3 ^(95% lymphocytes), a protein level of 0.72 g/l (normal range 0.15–0.45 g/l) and a glucose level of 2.3 mmol/l (blood glucose was 5.2 mmol/l). CSF culture and polymerase chain reaction analyses for herpes simplex virus, herpes zoster virus, enterovirus, Epstein Barr Virus and cytomegalovirus (CMV) were negative. The suspected diagnosis was AIDP, part of the heterogeneous GBS grouping.

He received supportive management and twice daily spirometry was undertaken. Although not dyspnoeic, his FEV_1 _was reduced at 2.7 l (59% of predicted) and oxygen therapy was commenced. Transfer to the Intensive Therapy Unit was planned if the FEV_1 _fell below 1.5 l. After neurological advice, a 5 day course of daily intravenous immunoglobulin therapy (IVIg) (0.4 g/kg/day) was commenced. Over the next 3 days his condition deteriorated and by day 4 of IVIg therapy, he had lost all lower limb power and was developing sensory abnormalities, upper limb weakness, difficulty swallowing and blunting of speech.

He had been successfully treated for syphilis in 2005. HIV antibody testing at that time was negative but, in view of his established risk factors and new symptoms, a repeat test was now undertaken and was positive. Screening for other blood-borne viruses, opportunistic infections and magnetic resonance imaging of brain and spine were negative.

**Figure 1:**
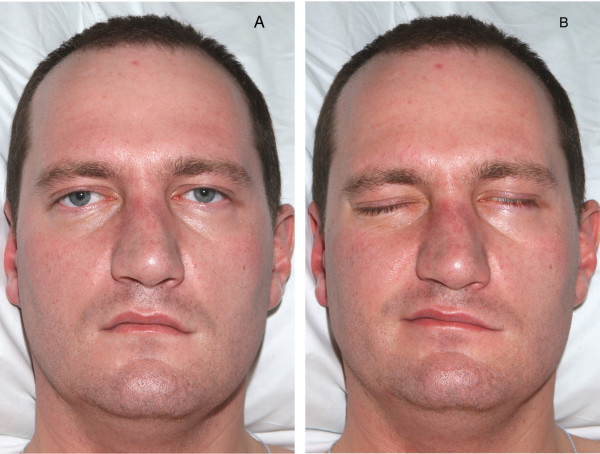
Lower motor neurone left sided facial nerve palsy demonstrated in photographs before (A) and after (B) patient attempts to close eyes and smile.

On day 5 of IVIg his speech was barely intelligible and left sided facial nerve palsy developed (Figure 1). Bilateral upper limb power was significantly reduced to MRC grade 1/5 but respiratory function remained adequate. His HIV viral load was >100,000 copies/ml and the CD4 count was 408 cells/mm^3^. He was commenced on highly active antiretroviral therapy (HAART) with zidovudine, lamivudine and efavirenz. Less than 12 hours later his symptoms plateaued and a gradual recovery began.

Sensory nerve conduction studies were normal. EMG motor studies revealed active denervation in distal lower limb muscles.

Over the next 9 weeks, he received intensive complex neuro-rehabilitation and at discharge no neurological deficits were present, but he continued to experience mild lower limb neuropathic pain. HAART stopped at 6 months with a CD4 count of 901 cells/mm^3^. He remains asymptomatic 7 months after withdrawal of HAART.

## Discussion

HIV infection is associated with a range of peripheral neuropathies; distal sensory polyneuropathy, mononeuritis multiplex, polyradiculopathy, demyelination and drug toxicities [1]. A specific association with GBS was first suggested in the 1980s. GBS remains uncommon in the general population and is a rare presentation of HIV, with only a small number of case reports in the literature. Retrospective case series from North America and sub-Saharan Africa suggest that GBS is more likely in acute HIV seroconversion or early infection [1,2]. These patients may be otherwise asymptomatic so it is vital to consider HIV testing in all previously well individuals with unusual neurological presentations. Our patient had a previously negative HIV test, a high viral load and a robust CD4 count, suggesting acute seroconversion, although this is impossible to conclusively prove as there are no blood samples from 2005–2007 to retrospectively test.

Guillain-Barré syndrome has also been described in advanced HIV. CMV polyradiculopathy can be clinically indistinguishable at CD4 counts <50 cells/mm [3], leading some authors to suggest that symptomatic individuals with advanced immunosuppression should also be treated presumptively for CMV [3].

Several pathological variants of GBS are recognised. AIDP represents 75% of cases and classically presents with ascending symmetrical paresis or paraesthesia, loss of deep tendon reflexes and autonomic dysfunction. Other subtypes include a purely motor variant called Acute Motor Axonal Neuropathy (AMAN), and Fisher's syndrome, characterised by acute ophthalmoplegia, ataxia and areflexia, often in association with lower cranial nerve palsies. Overlap syndromes combining the above features occur [4]. Our patient's presentation is typical of AIDP.

Diagnosis is based on clinical features, neurophysiological testing and lumbar puncture. The CSF protein level is raised in 80% of cases. CSF white cells are typically absent in HIV-negative patients but, as in our case, cells are regularly seen in HIV-GBS [5]. This distinction is not universal but further highlights the need for HIV testing when unexpected CSF findings are seen.

The pathogenesis of HIV-GBS is incompletely understood. Possible mechanisms include direct HIV neurotoxicity or autoimmunity. Several features favour an immune mechanism. Typical neural histology supports an antibody-mediated process and high titres of autoantibodies to myelin sheath glycosphingolipids are found in the serum of affected patients [3]. The presence of these anti-ganglioside antibodies at low CD4 counts suggests that abnormal immunoregulation in HIV may precipitate a paradoxical rise in autoantibodies, resulting in GBS.

In the initial stages, neurological weakness is rapidly progressive, involving respiratory muscles in 25% of cases. These patients may require mechanical ventilation. After a disease nadir, usually 2–4 weeks after onset of symptoms and a variable plateau phase, recovery occurs over a period of weeks to months. Mortality varies between four and 15%, and 20% remain disabled at one year despite treatment. Outcomes in HIV-positive patients are equivalent to those of HIV-negative patients. Early treatment with plasmapheresis or IVIg therapy have equal efficacy in reducing the proportion of patients requiring ventilation during the first four weeks [4].

Since 1996, HAART has revolutionised the treatment of HIV. Given the proposed role of autoimmunity in HIV-GBS, it seems likely that antiretroviral drugs will alter the course of neurological illness. Individual case reports of patients on pre-established HAART describe GBS as a drug side effect [6] or an immune reconstitution syndrome [7] but there are only five previously published cases of antiretroviral-naïve individuals presenting with HIV-GBS who received HAART as a component of acute management (Table 1) [8-11].

**Table 1:** Previously anti-retroviral naïve cases of HIV-GBS treated with HAART

Background/Demographics				Initial Treatment			Subsequent Progress
	
Reference	Age/Sex	GBS subtype	CD4 (cells/mm^3^)	IVIg given^#^	Mechanical Ventilation	First line HAART	
Bani-Sadr et al 2002 [9]	35/M	AIDP	149	Not stated	No	ZDV/3TC/IDV/RTV	Complete recovery, HAART continued

Gisslén et al 2005[6]	35/M	AIDP	914	No	No	d4T/3TC/SQV/NLF	Improvement at 3/12 & HAART stopped. GBS recurred 2/12 later. IVIg & 5/12 more HAART (same regimen). No further recurrence when HAART discontinued

De Castro et al 2006 [10]	38/M	AIDP	502	Yes	Yes	ZDV/3TC/IDV	Complete recovery on long-term HAART. 2 transient GBS recurrences during HAART interruption for unrelated toxicity; 1. Renal lithiasis at 18/12; IDV→RTV 2.GI upset at 31/12; wkly IVIg for 6/12 RTV→EFV

Wagner et al 2007 [11]	46/M	AMAN	150	No	No	Regimen not stated	Complete recovery, HAART continued

Hiraga et al 2007 [4]	56/M	Fisher/GBS overlap^+^	24	Yes	No	Regimen not stated	Complete recovery, HAART continued

Sloan et al 2008 [this paper]	30/M	AIDP	408	Yes	No	ZVD/3TC/EFV	Complete recovery, HAART stopped at 6/12

AZT: zidovudine, 3TC: lamivudine, IDV: indinavir, RTV: ritonavir, d4T: stavudine, SQV: saquinavir, NLF: nelfinavir, EFV: efavirenz.

# IVIg dose: 0.4mg/kg IV daily for 5 days

+ Patient also treated for CSF & Cryptococcal Antigen positive *Cryptococcus Neoformans* meningitis

Three antiretroviral-naïve HIV-GBS patients presented with low CD4 counts (<200 cells/mm^3^) and made a good recovery with long term HAART. Two others, like our case, began therapy at higher CD4 counts and improved. Our patient's apparent treatment response was unusually rapid, raising the possibility that HAART initiation was coincidental with onset of natural recovery but when viewed alongside the other cases it does seem that early introduction of antiretroviral treatment is beneficial, particularly if symptoms are severe and progressive.

Both previous cases of HIV-GBS at high CD4 counts encountered transient neurological relapses when HAART was stopped. Recurrent GBS or Chronic Inflammatory Demyelinating Polyneuropathy affects 3% of HIV negative patients [4], but has not been described with HIV. The duration of therapy at high CD4 counts and risk of relapse needs further study.

Finally, the choice of antiretroviral drug combination requires consideration. Eight drugs from three different antiretroviral classes were used in the five previous cases. The best combination is unclear. As HIV is a neurotropic virus and the nervous system is a sanctuary site for infection, drugs which effectively cross the blood-brain barrier are most appropriate. The nucleoside reverse transcriptase inhibitors zidovudine, lamivudine and abacavir, and non-nucleoside reverse transcriptase inhibitors efavirenz and nevirapine penetrate the central nervous system well but protease inhibitors are less reliable. This explains our choice of zidovudine, lamivudine and efavirenz. Nevirapine was avoided as the risk of adverse events with this drug increases at higher CD4 counts.

## Conclusion

The AIDP variant of GBS is a rare presentation of acute HIV seroconversion, with significant mortality and morbidity. Early neurological diagnosis and consideration of HIV testing are important to improve outcome. Although data are sparse, it appears reasonable to consider early commencement of HAART using drugs with good CNS penetration, particularly if there is continued deterioration on conventional therapy. This case adds to the limited literature on this topic and highlights the need for further study.

## Abbreviations

HIV: Human immunodeficiency virus; GBS: Guillain-Barré syndrome; AIDP: Acute inflammatory demyelinating polyneuropathy; IVIg: Intravenous immunoglobulin; HAART: Highly active antiretroviral therapy; CNS: central nervous system; CSF: cerebrospinal fluid; MRC: Medical Research Council; CMV: cytomegalovirus; FEV_1_: Forced expiratory volume in one second; EMG: Electromyography; AMAN: Acute motor axonal neuropathy

## Consent

Written informed consent was obtained from the patient for publication of this case report and accompanying images. A copy of the written consent is available for review by the Editor-in-Chief of this journal.

## Competing interests

The authors declare that they have no competing interests.

## Authors' contributions

DS and MBJB conceived of and drafted the case presentation. DS reviewed the literature and wrote the discussion and conclusions. AN provided specialist neurological input. AROM and NJB were the consultants responsible for the patient's care and provided general support. All authors read and approved the final manuscript.
